# Acidic microenvironment responsive polymeric MOF-based nanoparticles induce immunogenic cell death for combined cancer therapy

**DOI:** 10.1186/s12951-021-01217-4

**Published:** 2021-12-28

**Authors:** Xiaoli Zhang, Yi Lu, Die Jia, Wei Qiu, Xianbin Ma, Xingliang Zhang, Zhigang Xu, Feiqiu Wen

**Affiliations:** 1 Pediatric Research Institute, Department of Hematology and Oncology, Shenzhen Children’s Hospital, Shenzhen, 518038 Guangdong People’s Republic of China; 2 School of Materials and Energy and Chongqing Engineering Research Center for Micro-Nano Biomedical Materials and Devices, Southwest University, Chongqing, 400715 People’s Republic of China

**Keywords:** Nanoreactor, Tumor-specific activatable, Synergistic therapy, Immunogenic cell death, Multi-modal imaging

## Abstract

**Background:**

The complex tumor microenvironment and non-targeting drugs limit the efficacy of clinical tumor therapy. For ensuring the accurate delivery and maximal effects of anticancer drugs, it is important to develop innovative drug delivery system based on nano-strategies.

**Result:**

In this study, an intracellular acidity-responsive polymeric metal organic framework nanoparticle (denoted as DIMP) has been constructed, which can co-deliver the chemotherapy agent of doxorubicin (DOX) and phototherapy agent of indocyanine green (ICG) for breast carcinoma theranostics. Specifically, DIMP possesses a suitable and stable nanometer size and can respond to the acidic microenvironment in cells, thus precisely delivering drugs into target tumor sites and igniting the biological reactions towards cell apoptosis. Following in vivo and in vitro results showed that DIMP could be effectively accumulated in tumor sites and induced powerful immunogenic cell death (ICD) effect.

**Conclusion:**

The designed DIMP displayed its effectiveness in combined photo-chemotherapy with auxiliary of ICD effect under a multimodal imaging monitor. Thus, the present MOF-based strategy may offer a potential paradigm for designing drug-delivery system for image-guided synergistic tumor therapy.

**Graphical Abstract:**

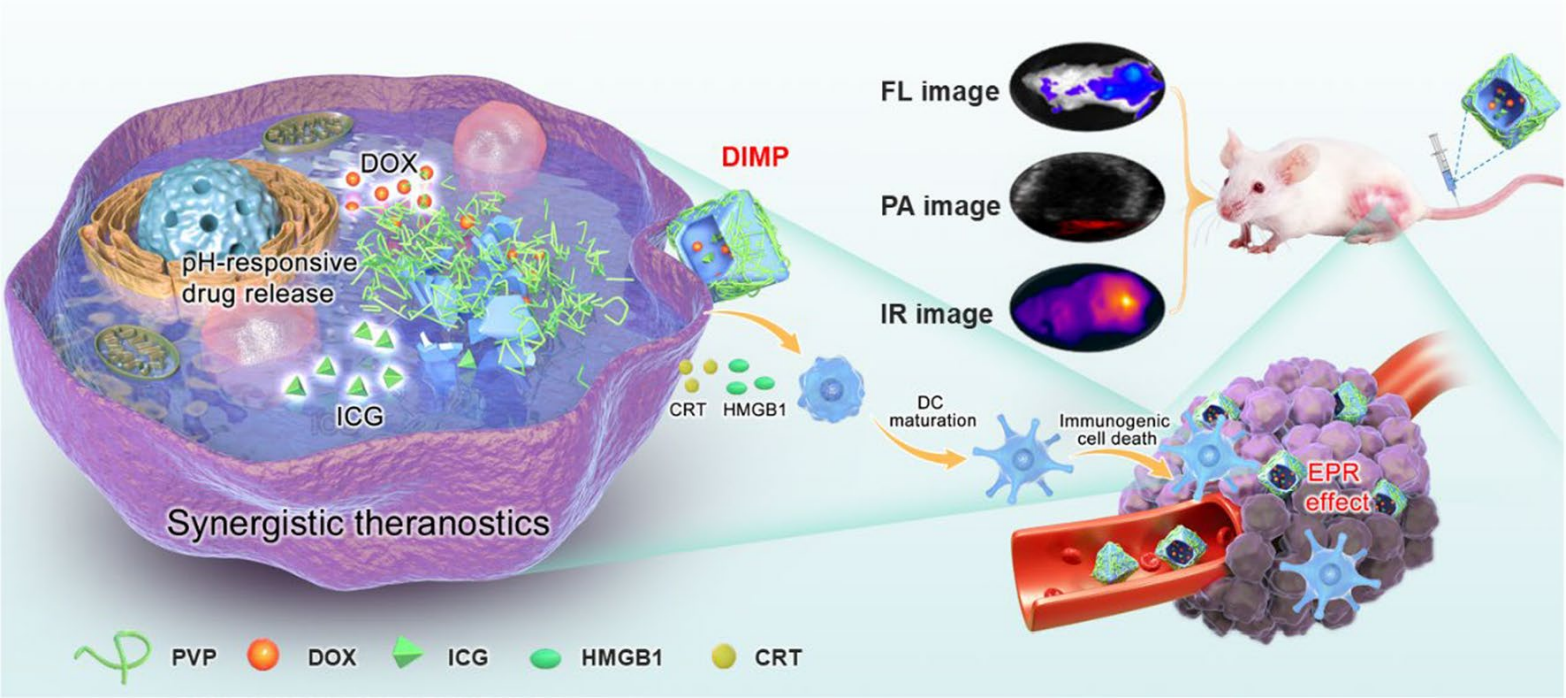

**Supplementary Information:**

The online version contains supplementary material available at 10.1186/s12951-021-01217-4.

## Introduction

Cancer poses a serious threat to public health, and exploring innovative iatrotechnics of cancer treatment has been a consensus for anti-cancer cause [1]. At present, chemotherapy still dominates in the treatment of various cancers. However, traditional chemotherapy has certain limitations, such as severe side effects, poor pharmacokinetics, and the lack of targeting [2]. Recently, a variety of synergistic therapies have been developed based on chemotherapy, which can maximize the efficiency of tumor treatment, such as the combined chemotherapy and photodynamic therapy (PDT) [3]; chemotherapy and photothermal therapy (PTT) [4]; chemotherapy and photoacoustic therapy [5, 6] and so on. The synergistic therapies can combine the advantages of various therapies, thus reducing the side effects and promoting the therapeutic efficiency. For instance, PTT can increase the temperature of tumor site [7, 8] and enhance the vascular permeability, which is conductive to accumulate the chemotherapy drugs at the tumor site. Meanwhile, PDT has the advantages of fast, accurate, reproducible and minimal side effects [9, 10]. Therefore, it is of great significance to develop a synergistic therapy combing chemotherapy, PTT and PDT for the treatment of cancer [11, 12].

The development of nanoplatform has realized the design and construction of synergistic therapy. In the past few decades, researchers have developed various nanocarriers, such as inorganic mesoporous silica [13, 14], metal nanoparticle (NP) [15, 16], liposomes [17, 18] and organic micelles [19, 20]. The ideal drug-loading system should have the following advantages: high drug loading rate and permeability [21], appropriate nanometer size, and good biocompatibility. Metal–organic framework (MOF) is a new and potential material as nanocarrier [22–26]. It has several merits including adjustable nanometer size, diverse functionalization, high drug loading, good biocompatibility and biodegradability. In short, these ideal properties make MOF a promising platform for drug delivery and clinical tumor therapy.

In the application of nanocarrier-based drug delivery system, it is important to perform the real-time monitor on the enrichment and therapeutic effects of drugs at the tumor site, which is beneficial to improve the efficiency of tumor treatment [27]. Indocyanine green (ICG) is a water-soluble compound as a common contrast agent for near-infrared (NIR) fluorescence imaging. Since enrichment in tumor tissues, ICG can serve as a NIR fluorescence/photoacoustic dual-modal imaging agent in the field of disease diagnosis [28–30]. In clinical practice, the tumor location and boundary can be accurately figured through ICG. At the same time, NIR light is efficiently captured by ICG molecules and converted into the heat and singlet oxygen to attack tumor cells. However, ICG can be quickly cleared out by the circulatory system of liver. Due to high metabolic rate in the body and low cell uptake property, the application of ICG is limited. In recent years, ICG has been frequently exploited as one component of multifunctional nanodrug delivery systems in the precision diagnosis and treatment of malignance [31–33]. As an excellent chemotherapy drug, Doxorubicin (DOX) is meaningful in the treatment of various cancers, which uses alone or in combination with other drugs in the clinic [34–36]. DOX kills tumor cells by inhibiting the synthesis of RNA and DNA (making the strong inhibitory effects on RNA). However, the free anti-tumor effect of DOX is restricted to weak penetration ability in the tumor so that DOX cannot reach the tumor site accurately and own toxic side effects on normal cells. Therefore, the design of drug delivery system integrated both ICG and DOX has been significant in clinical applications. Meanwhile, nanocarrier-based drug delivery system has verified by researcher as the effective inducer for immunogenic cell death (ICD) [37, 38], which can accelerate the mature of dendritic cells (DCs) and improve the therapeutic effect. DOX is a classical ICD agent while ICG-based phototherapy also shows a strong ICD effect [39, 40], where it is reasonably assumed that synergistic chemotherapy-phototherapy can be served as promising strategy for enhancing tumor therapy by the induce of ICD process.

**Scheme 1:**
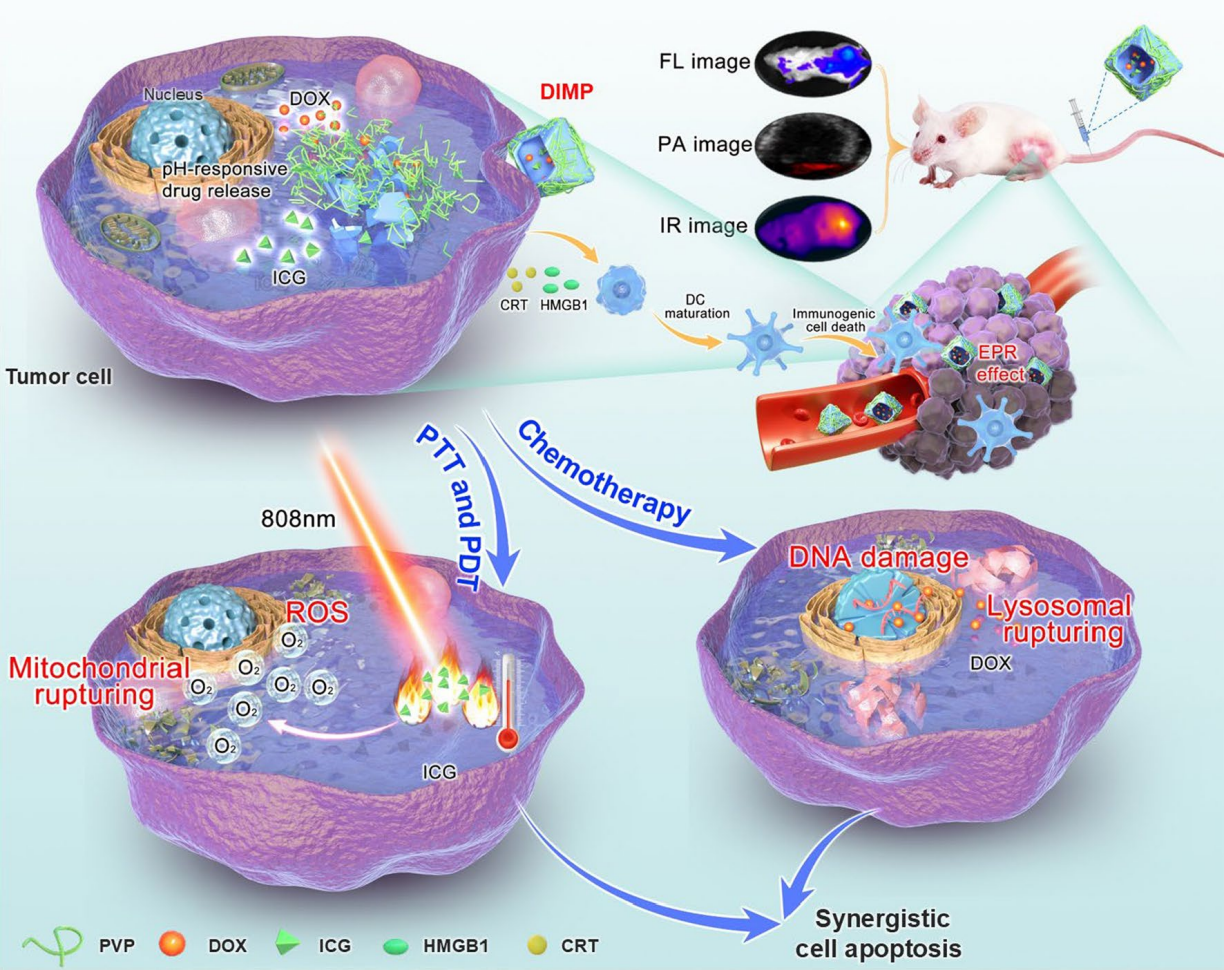
Schematic illustration of the synthetic strategy of DIMP and the IR/PA/FL imaging-mediated combined cancer therapy

Considering all above factors, we have constructed a MOF-based nanoreactor of DIMP for achieving multi-modal imaging guided chemotherapy/PTT/PDT (Scheme 1). The obtained DIMP have a high drug loading capacity and good stability under physiological conditions. Meanwhile, DIMP can be effectively accumulated at the tumor site, and then controllably liberate drugs obeying a pH-triggered model. In addition, the photosensitizer of ICG encapsulated in DIMP can be used for photoacoustic (PA) imaging/infrared thermal imaging (IR)/Fluorescence image (FL), which can bring a real-time monitoring for the therapeutic process. The systemic in vivo and in vitro results demonstrate that DIMP can generate strong immunogenic cell death effect and exhibit excellent therapeutic effectiveness through a synergistic chemotherapy/PTT/PDT therapy against tumors. In short, a MOF-based nanoreactor has been designed, which brings a new approach to realize the multimodal tumor theranostics.

## Materials and methods

### Materials

Zinc nitrate hexahydrate (Zn(NO_3_)·6H_2_O, 99.5%) was supplied by Macklin, 2-methylimidazole (98%), doxorubicin hydrochloride (DOX·HCl, 98%), Cardio-Green (ICG, 95%), methanol (99.5%) and polyvinylpyrrolidone (PVP) were purchased from Sigma-Aldrich. All agents related cell assays were supplied by Life Technologies Corporation. Deionized water was prepared by a Milli-Q Synthesis A10 System.

### Preparation of MOF@ICG@DOX (DIMP)

1.2 mmol of 2-methylimidazole and 0.2 mmol of zinc nitrate hexahydrate were solved in 5 mL of methanol and then stirred for 5 min at 25 °C. After the mixture was fully dissolved, 3 mg of DOX and 1 mg of ICG were added. The reaction flask was sealed and protected from light for 2 h. After the reaction was completed, the solution was transferred to a 15 mL centrifuge tube and centrifuged at 8000 rpm for 10 min in a high-speed centrifuge. After the supernatant was removed, 5 mL of DI water was added in the centrifuge tube for further centrifuging. Above operation was repeated for three times. The pellet after centrifuging was re-dispersed in 10 mL of DI water, and then 3% PVP was added for 12 h stirring. Finally, the mixture was centrifuged for removing unreacted PVP, and the obtained DIMP NPs were redispersed in water for further experiment.

### Drug release from DIMP

1.0 mL of DIMP was put in a dialysis bag (MW: 3500), and then it was immersed in 80 mL of phosphate buffered saline (PBS) with different pH values. After shaking for 48 h at 37 °C, 1 mL of dialysate was taken out at the predetermined time point for determining the amount of released drug, and fresh PBS with corresponding pH value was added. As described above, the concentration of DOX in the sample was measured by fluorescence spectrometer.

### Photothermal effects of DIMP

Different concentrations of DIMP NPs (0, 5, 10, 20 and 40 μg/mL) was put into a 2 mL centrifuge tube. The centrifuge tube was irradiated with 808 nm laser and the temperature change of DIMP were recorded by thermal imager (FLIR E95) every 1.5 min within 6 min. The photothermal effect of DIMP was verified. Firstly, the irradiating time of DIMP NPs with different concentrations was prolonged at a fixed power of 1.5 W/cm^2^. The temperature change was recorded once every minute within 15 min for plotting the heating curve. The DIMP NPs of 40 μg/mL was selected as the optimal concentration. Then, the concentration of DIMP NPs was fixed at 40 μg/mL, and the temperature change under the irradiation of 808 nm laser was recorded with different powers (0, 0.5, 0.8, 1.5 and 2.0 W/cm^2^). Next, the photothermal conversion efficiency of DIMP NPs was verified, three consecutive On–Off cycles of DIMP NPs (40 μg/mL) under laser irradiation (1.5 W/cm^2^) were recorded.

### In vitro anti-tumor activity

The in vitro cytotoxicity of DIMP was studied by methylthiazolyl tetrazolium (MTT) method. 4T1 cells (with a density of 1 × 10^4^ cells/well) were planted in 96-well plate. Then, DOX, ICG + L, DIMP, and DIMP + L (L: with laser) were used to incubate with the cells for 6 h. The DOX concentration was 0, 0.01, 0.5, 1.0 and 5.0 μg/mL, and the corresponding ICG concentration was 0, 001, 0.03, 0.17, 0.3 and 1.7 μg/mL. Then the old medium was replaced with fresh medium and further radiated by 808 nm laser (1 W/cm^2^) for 5 min. After the laser treatment, the cells were incubated for another 18 h. After the drug was applied for 24 h, the culture medium was discarded. Then, the cell viability was determined with MTT. The absorbance at 490 nm was measured by the microplate reader.

Live/dead cell co-staining experiments were used to test killing ability of DIMP to tumor cells. First, 4T1 cells (with a density of 1 × 10^5^ cells/well) were seeded into 12-well plate. After the cells adhered to the wall, the 1640 medium containing DOX, ICG and DIMP were added, respectively. After 6 h of drug treatment, the old medium was replaced with the new medium, and the cells were incubated for 18 h with or without laser. Then the cells were stained with live/dead kit and imaged with fluorescence microscope (Olympus-IX73).

### Cellular uptake

4T1 cells were transferred into a 12-well plate, and then adhered to the wall. The old medium was replaced with the medium containing DIMP (equal to 15 μg/mL of DOX). After incubating for 2 h and 6 h, the cells were washed with PBS and fixed with 4% paraformaldehyde solution for 30 min. Then the cells were washed with PBS again, Triton X-100 was added for 5 min and BSA was incubated for 30 min. The cell nuclei were stained with DAPI. Finally, the cells were observed with confocal laser scanning microscope (CLSM, Zeiss-8000). Next, 4T1 cells in 24-well plate were incubated for 12 h, and then 1640 medium containing DIMP (8 μg/mL of DOX) was added for 0.5, 1.0, 2.0, 4.0 and 6.0 h, respectively. Then the cells were washed with PBS and centrifuged for following flow cytometry analysis.

### Generation and detection of reactive oxygen species (ROS)

1,3-Diphenylisobenzofuran (DPBF) was applied to evaluate the ROS (^1^O_2_) generation ability of DIMP. First, 5 μL of DPBF in dimethylsulfoxide (DMSO) solution (10 mmol) was added to 1 mL DIMP solution (10 μg/mL of ICG), and then the mixed solution was illuminated with laser (808 nm, 1 W/cm^2^). At regular time points, the change of UV–Vis absorption spectrum of DPBF was observed at 410 nm for determining the production of ^1^O_2_.

The single oxygen sensor green (SOSG) was applied as high sensitive fluorescent probe for the detection of ^1^O_2_ and photo-generated singlet oxygen. Firstly, the 4T1 cells were incubated with medium containing DOX, ICG and DIMP for 6 h, and then the SOSG (2.5 μM) was added for the laser irradiation (808 nm, 1 W/cm^2^, 5 min). The fluorescence of SOSG excited at 504 nm was measured. Images were obtained using a confocal fluorescence microscope.

### Cell colocalization analysis of DIMP

4T1 cells (with the density of 4 × 10^4^ cells/well) were added to a 12-well plate and incubated with DIMP (DOX: 15 μg/mL) for 2 h and 6 h, respectively. Then, Mito-Tracker™ orange (Mito-Or) and Lysosomal Blue DND-22 (Lyso-Blue) were used to stain cell mitochondria and lysosomes. The cell colocalization images were obtained with CLSM.

### The transformation of cellular mitochondrial and lysosomal membrane

Since mitochondria were closely related to cell apoptosis. Once mitochondrial transmembrane potential collapsed, cell apoptosis was irreversible. Therefore, the JC-1 probe was used to study the effects of DIMP on membrane potential of mitochondria. 4T1 cells (with the density of 4 × 10^4^ cells/well) were seeded on a 12-well plate. Cells were incubated with fresh medium containing DIMP for 6 h, and then washed with PBS and supplemented with fresh medium. The cells were treated with different methods, with or without light illumination (808 nm, 1 W/cm^2^, 5 min). Finally, cells were stained with JC-1 for 20 min and then imaged with CLSM.

4T1 cells (with the density of 4 × 10^4^ cells/well) were seeded into the 12-well plate. The cells were incubated in the medium containing ICG and DIMP (equal to 15 μg/mL of DOX) for 6 h, the medium was replaced with fresh medium without phenol red. The cells were irradiated for 5 min with or without laser radiation, and then the cells were incubated for 2 h. Finally, the cells were stained with AO (5 μm) and observed with CLSM.

### DNA damage

4T1 cells (with the density of 5 × 10^4^ cells/well) were added to a 12-well plate, and were incubated at 37 °C and 5% CO_2_ for 24 h. The cells were divided into five groups: (1) control group (control), (2) ICG with light (ICG + L), (3) free DOX (DOX), (4) DIMP (DIMP), (5) DIMP with light (DIMP + L). The medium containing ICG, DOX and DIMP (DOX: 10 μg/mL) was added into the petri dish. After incubating for 24 h, the cells were irradiated with laser (808 nm, 1 W/cm^2^, 5 min). After 24 h, the cells were fixed with 4% paraformaldehyde and infiltrated with 1% Triton X-100. Then, the cells were treated with 1% bovine serum albumin (BSA) for another 1 h to prevent non-specific protein interactions. The cells were treated with anti-γ-H2AX mouse monoclonal antibody (ab81299, Abcam Inc.) at 4 °C overnight. Finally, the secondary antibody (goat anti-rabbit IgG) was added and incubated for 1 h, and Hoechst 33342 was applied for staining the nuclei for 10 min. The cells were observed with CLSM.

### Immunologic cell death (ICD) assay in vitro and in vivo

The ICD effect induced by DIMP was assessed via a classical detection of high-mobility group box 1 (HMGB1) and calreticulin (CRT) in cellular and mice levels. Firstly, a dish with 5 × 10^4^ 4T1 cells were incubated overnight and then treated with PBS, ICG + L, DOX, DIMP and DIMP + L for 24 h. After washed by PBS and blocked process by 10% normal goat serum, the cells were treated with primary antibodies of HMGB1 or CRT at 4 °C for 12 h. Then the cells were further treated with secondary antibody-647 for 30 min and nuclear staining by DAPI for 10 min. Finally, the immunofluorescence images were recorded by CLSM. Furthermore, we detected the in-vivo ICD immunofluorescence effect in tumor sections. After the end of treatment in mice model, tumors from each group were sliced into sections, which were sequentially treated with primary antibody, secondary antibody and DAPI staining, and the results were obtained using CLSM.

### Tumor penetration in vitro

Multicellular spheroids (MCSs) tumors induced by 4T1 cells were used to study the in vitro tumor penetration behavior of DIMP. First, 100 μL of 1% agarose was added to a 96-well plate, then 4T1 cells were inoculated into 96-well plate, and MCSs were successfully formed after 5 days of incubation. The old medium was removed and the fresh medium containing DIMP (equal to 15 μg/mL of DOX) was added. The MCSs were incubated for 8 h, and the penetration ability was checked by CLSM. 

### Animal models

The mice used in the experiment were Balb/c mice (6–7 weeks old, about 18 g), and all animals were from Chongqing Tengxin Biotechnology Co., Ltd. All animal experiment protocols strictly abide by the National Laboratory Animal Care and Use Guidelines, and are approved by the Institutional Animal Care and Use Committee (IACUC) of Southwest University. The 4T1 mice breast cancer model was established by subcutaneous injection of 5 × 10^6^ cells.

### Animal imaging

200 μL of PBS, ICG and DIMP (2.5 mg/kg) were injected into the tail vein respectively. After 24 h, certain intensity of irradiation (808 nm, 1 W/cm^2^) was performed. Then, the images were taken at the specified time point by using the infrared camera (tis55, fluke). The PA performance of DIMP in vivo was evaluated by Vevo LAZR Photoacoustic Imaging System (Visual Sonics Inc, Toronto, Canada). 200 μL of DIMP (4 mg/mL) was injected via tail vein, and PA images were collected at 0 and 24 h after injection.

Next, the Balb/c mice were split into two groups (three in each group). 150 μL of ICG and DIMP (both containing 55 μg/mL ICG) were injected via tail vein. At 12, 24, 48 h after injection, fluorescence imaging images were obtained by using the near infrared (NIR) imaging system (PerkinElmer IVIS lumina kinetic Series III). The mice were sacrificed after 48 h injection, and organs including the heart, liver, spleen, lung, kidney and tumor were collected for imaging and analysis of bio-distribution.

### In vivo anti-tumor efficacy evaluation

When the tumor volume reached about 100 mm^3^, the mice were divided into the following five groups (five in each group): (1) Control (PBS); (2) DOX; (3) ICG + L; (4) DIMP; (5) DIMP + L (L: with light illumination). 150 μL of PBS, DOX, ICG and DIMP (including 5 kg/mL of DOX and 4 kg/mL of ICG) were injected into the tail vein, respectively. After administration for 24 h, the tumors of mice were irradiated with 808 nm laser (1 W/cm^2^, 5 min). The tumor volume and weight of each mouse were recorded every day during the treatment. After 12 days of treatment, the mice were sacrificed and the tumor, heart, liver, spleen, lung and kidney tissues were collected and fixed with 4% paraformaldehyde for 24 h. The samples were embedded in paraffin and cut into thin slices. The fluorescence of drug in tumor sections was observed with CLSM, so as to evaluate the penetration and enrichment ability of DIMP in tumor sites. The fixed slices were stained with hematoxylin and eosin (H&E) and terminal deoxynucleotidyl transferase mediated dUTP biotin nick end labeling (TUNEL) to study the histo-toxicity or apoptosis of tumor cells. The Ki67 staining was also performed to explore the proliferation of tumor cells.

### Blood routine test

In order to evaluate the potential toxicity of DIMP, the whole blood of mice after administration was analyzed. Balb/c mice were divided into four groups (3 in each group) were injected with Control (PBS), DOX, ICG and DIMP through the tail. Seven days later, blood from the eye socket was obtained for test. Blood routine included white blood cell count (WBC), red blood cell count (RBC), hematocrit (HCT), mean corpuscular hemoglobin concentration (MCHC), lymphocyte ratio (LYM), coefficient of variation of red blood cell distribution width (RDW), hematocrit (HCT), hemoglobin concentration (HGB), mean corpuscular hemoglobin concentration (MCHC) and variation of red blood cell distribution width (RDW), blood platelet counts (PTL) and mean platelet volume (MPV) were measured. Blood routine was performed by blood analyzer (Mindray bc-2600vet). At the same time, the main organs (heart, liver, spleen, lung and kidney) of mice were collected, and the corresponding tissue slices were stained with H&E to detect the potential toxicity of DIMP.

### Statistical analysis

All data were expressed as mean ± standard deviation (SD) and the significant difference of the results was evaluated by *t-test* and analysis of variance.

## Results and discussion

### Characterization of physicochemical properties of DIMP NPs

**Fig. 1:**
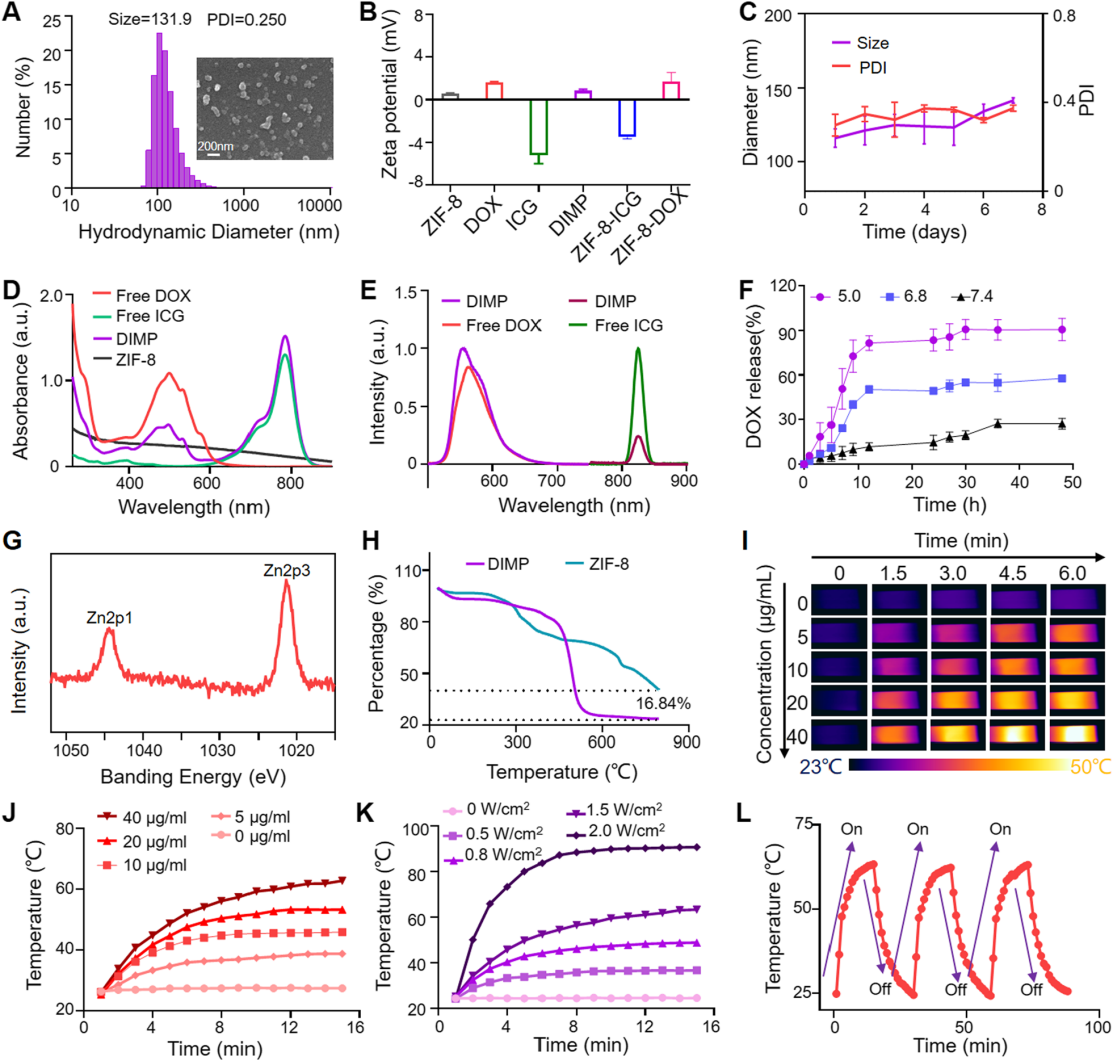
**A** The morphology and size distribution of DIMP. **B** Zeta potential of ZIF-8, DOX, ICG and DIMP, and data were expressed as mean ± SD (n = 3). **C** Diameter distribution and the PDI change of DIMP under different incubation time. **D** UV–Vis absorption spectrum and **E** Fluorescence emission spectrum of DOX, ICG and DIMP. **F** The release curve of DOX under different pH conditions. **G** XPS patterns of the DIMP. **H** TGA curves of DIMP and ZIF-8. **I** Infrared thermal image of DIMP with different concentration and under different irradiation time of the 808 nm laser (1.5 W/cm^2^). **J** Photothermal heating curves of DIMP with different concentration under NIR laser (808 nm, 1.5 W/cm^2^). **K** Photothermal heating curve of DIMP with the same concentration and under different power of NIR laser (808 nm). **L** The photothermal recycle curve of DIMP

DIMP NPs were obtained through a series of reactions and the particle size was determined with particle size analyzer. From Fig. 1A, the size was 131.9 nm, and the polymer dispersity index (PDI) was about 0.25, indicating that DIMP NPs had suitable particle size and uniform dispersion in aqueous solution. The scanning electron microscope (SEM) showed that the morphology of DIMP NPs was irregular, nearly round, with the particle size of about 100 nm. The larger hydrated particle size of DIMP obtained with dynamic light scattering (DLS) may be caused by swelling in the aqueous solution.

As show in Fig. 1B, the potential of ZIF-8 was 0.65 mV, and the potential after loading DOX and ICG was 0.86 mV, which was conducive to the drug circulation in the blood. The stability of the DIMP was monitored (Fig. 1C), and the particle size and PDI were measured and recorded for 7 days, suggesting good stability. UV–Vis spectrophotometer (Fig. 1D) and fluorescence spectrophotometer (Fig. 1E) were applied to measure the optical properties of DIMP. The UV results showed that the DOX and ICG peaks in DIMP were at 488 nm and 780 nm, respectively, which were corresponding to the free drugs. The fluorescence results showed that the peaks of DOX and ICG in DIMP were at 550 nm and 820 nm, which were also consistent with the peak positions of free drugs. These results collectively indicated that two drugs were successfully loaded on ZIF-8.

Furthermore, the lysosome in cells showed an acidic condition. In order to study the release behavior of DIMP in tumor cells, the in vitro release ability of DOX in DIMP was explored under different pH values (Fig. 1F). The release kinetics of DOX was investigated by measuring the fluorescence at 535 nm. The DOX release rate of the DIMP system was 27.3% at pH = 7.4, which was not obvious. At pH = 6.8, the content of DOX was increased in the buffer, reaching a release rate of 57.7%. When pH = 5.0, the DOX release rate was 90.7%. The acid-responsive DIMP was beneficial to chemotherapy, because imidazole can be easily decomposed when exposed to acid. It indicated that DIMP could efficiently decompose and release chemotherapeutic drugs in the TME, thus improving the efficacy of tumor treatment.

The crystal structure of DIMP was verified by XRD (XRD-700). As shown in Additional file 1: Fig. S2, the characteristic peaks of ZIF-8 were at 7.6°, 10.5°, 13°, and 18.5°, respectively. And DIMP also had the same characteristic peak in the corresponding position, which showed that DIMP had the crystalline form of ZIF-8. The elements in DIMP was analyzed with scanning results of XPS. As shown in Additional file 1: Fig. S2, the photon lines at the binding energy of about 270, 400, 520, and 1020 were attributed to N1s, C1s, O1s, and Zn2p, respectively. Among them, the Zn2p spectrum was shown in Fig. 1G, and the peaks of Zn–O were at 1021.6 and 1044.7 eV. The DOX and ICG (wt%) of DIMP grafted on ZIF-8 surface was evaluated with thermogravimetric analysis (TGA) (Fig. 1H). ZIF-8 NP was thermally stable at temperature as high as 300 °C, while DIMP tended to degrade at around 200 °C with a weight loss of about 16.84%.

### Characterization of photothermal properties of DIMP

In order to observe the photothermal effects of the DIMP, the temperature changes were recorded with thermal imager. After irradiation, the temperature changes of DIMP with different concentrations (0, 5, 10, 20 and 40 μg/mL) were recorded every 1.5 min within 6 min. From Fig. 1I, the temperature of DIMP with a concentration of 40 μg/mL can eventually reach about 50 °C.

The irradiation time was extended for 15 min to verify the temperature change curve of DIMP NPs in different concentrations. In Fig. 1J, the temperature of DIMP NPs at various concentrations was gradually increased under the irradiation of 1.5 W/cm^2^ 808 nm laser, in which it can exceed 60 °C at the maximum concentration of 40 µg/mL. The temperature of the PBS group did not change significantly. Further, DIMP remained a stable temperature without light. As the power gradually increased, DIMP NPs show different tendency (Fig. 1K). At 1.5 W/cm^2^, the temperature was around 60 °C. When it was increased to 2 W/cm^2^, it only took 8 min, and the temperature was already close to 90 °C. Then the On–Off effect of the DIMP NPs was verified (Fig. 1L), where it could remain unchanged after three consecutive laser On/Off cycles, showing an excellent thermal stability.

### Cell up-taking and cell viability

**Fig. 2:**
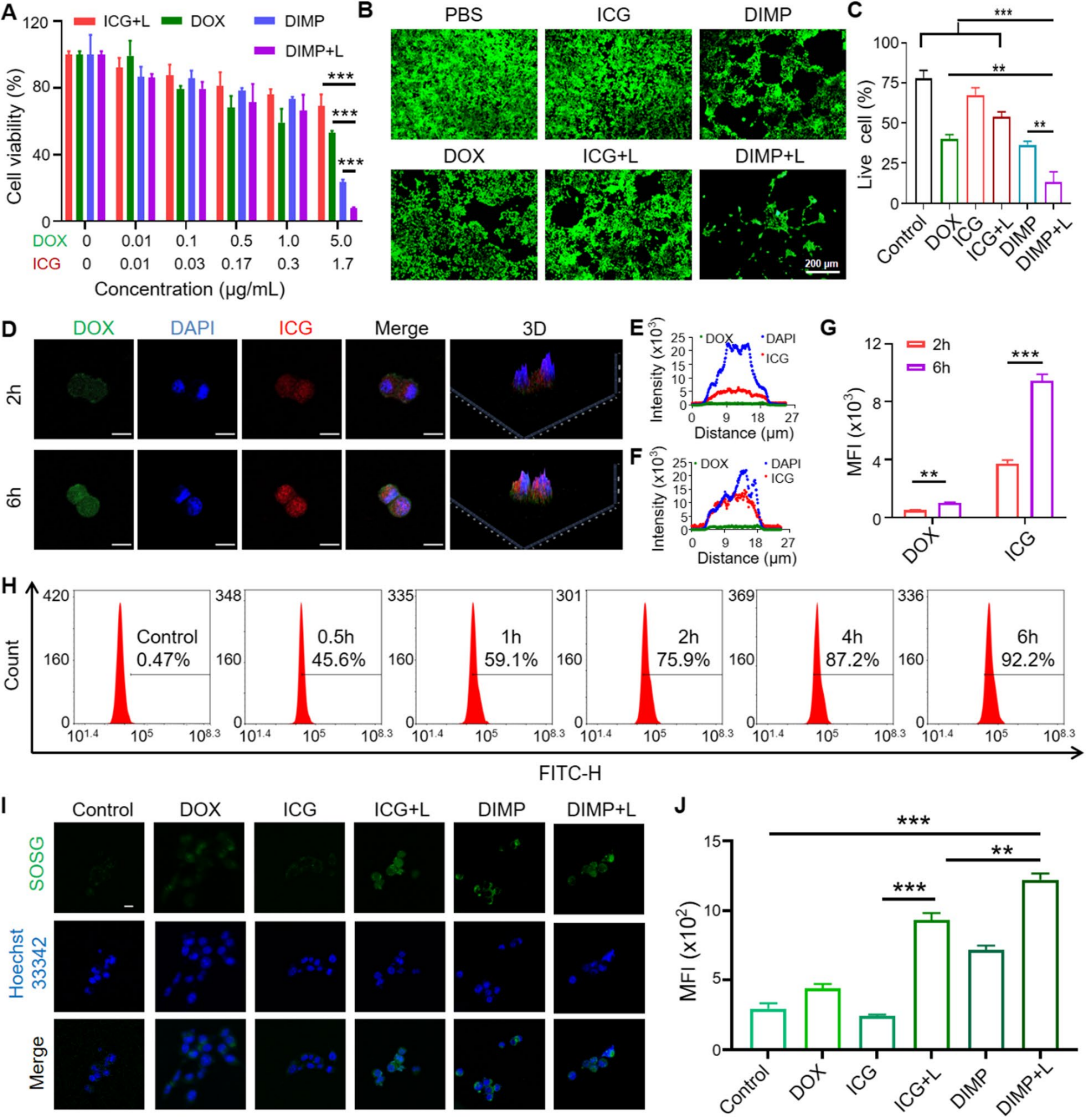
**A** The survival rate of 4T1 cells after the treatment with different concentrations of DOX, ICG and DIMP for 24 h. **B** Live/dead assay of cells and **C** quantification results of living cells. **D** Confocal fluorescence imaging showed the uptake of DIMP in 4T1 cells (Scale bar: 10 μm). The distribution curves (**E**, **F**) and mean fluorescence intensity (MFI) of DOX and ICG at 2 h and 6 h, respectively (**G**). **H** Flow cytometry analysis of the cellular uptake of DIMP for 0.5 h, 1 h, 2 h, 4 h and 6 h. **I** ROS generation in 4T1 cells incubated with ICG, DOX and DIMP with or without laser irradiation and **J** the results of average fluorescence intensity. (Scale bar: 20 μm). The data showed mean ± SD (n = 3). *: P < 0.05, **: P < 0.01 and ***: P < 0.001

The toxicity of DIMP and free drugs was determined with MTT. As shown in Fig. 2A, the toxicity of DOX was dominant at low concentrations. However, when the concentration was increased to 5 µg/mL, the toxicity of the DIMP group was much higher than that of the free drug group, with a significant difference. The cell viability of the DIMP + L group was less than 10%.

The cytotoxicity was further verified with live cell staining. The cells were photographed with a fluorescence microscope and quantified with Image J (Fig. 2B). The number of cells in the DIMP group was smaller, and the image obtained in DIMP + L group was significantly different from other control groups (Fig. 2C). Above results indicated that the DIMP group exhibited high cytotoxic effects under 808 nm laser.

To further study the ability of the drug to enter cells, the fluorescence intensity of 4T1 cells after treating with DIMP for 2 h and 6 h was observed by CLSM (Fig. 2D). The fluorescence of DOX and ICG was increased significantly with the lasted time (Fig. 2E, F). In order to accurately control the fluorescence intensity, the measurement tool of CLSM was applied for quantification. As shown in Fig. 2G, there was a significant difference in mean fluorescence intensity (MFI) between the treatment of 2 h and 6 h. Flow cytometry was applied in exploring the drug internalization. The drug intake was significantly increased over time, with cell phagocytosis exceeding 50% at 1 h and over 90% at 6 h, indicating that DIMP could be quickly ingested by cells (Fig. 2H).

### In vitro photodynamic effects

1, 3-Diphenylisobenzofuran (DPBF) was used as a singlet oxygen capture agent, and the relative consumption of DPBF under light condition was measured with ultraviolet–visible spectrophotometer. In the DPBF solution containing DIMP, under 808 nm laser irradiation, the UV absorption intensity of DPBF continued to decrease with the increase of the irradiation time (Additional file 1: Fig. S3), indicating that the consumption of DPBF increased with the increase of the irradiation time, to manifest the existence of singlet oxygen. The experimental results proved that DIMP had excellent active oxygen generation ability. The ROS production of DIMP was demonstrated with the fluorescent probe SOSG. As shown in Fig. 2I, the green fluorescence of the control groups was negligible without laser irradiation, and the green fluorescence of 4T1 cells incubated with DMIP was not obvious in the dark. However, when the 4T1 cells were irradiated with 808 nm laser (1 W/cm^2^) for 5 min, green fluorescence was obvious. The average fluorescence intensity of SOSG in the cells was shown in Fig. 2J. All these results indicated that DMIP had a strong ability to produce ROS, which had a promising application in PDT.

### Lysosomes and mitochondria co-localization assay

As an important processing plant for cells, lysosome was important organelles for the study of drug internalization pathways. As shown in Additional file 1: Fig. S4, the fluorescence intensity of DOX and ICG at 6 h was significantly stronger than 2 h. The Mander overlap coefficient (R) shows that DOX increased from 0.55 to 0.84, and ICG increased from 0.33 to 0.89. The above results indicate that DIMP has a good co-localization effect.

**Fig. 3:**
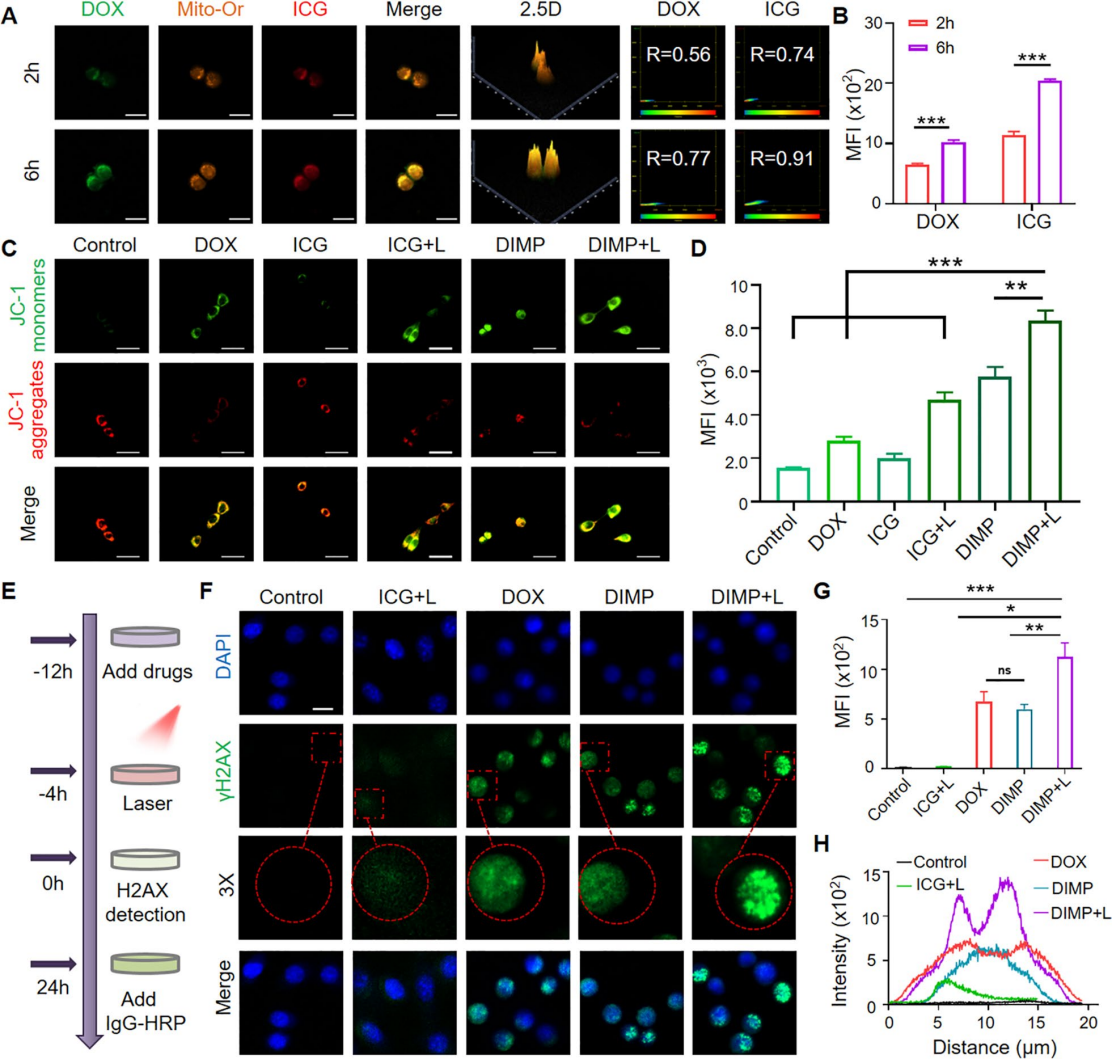
**A** The 4T1 cells were cultured for 2 h and 6 h with DIMP was observed with laser confocal microscopy. **B** The average fluorescence intensity of DOX and ICG was quantified (Scale bars: 20 μm). **C** 4T1 cells were incubated with DIMP for 6 h and stained with JC-1. Red fluorescence indicated JC-1 aggregates, green fluorescence indicated JC-1 monomers (Scale bar: 50 μm). **D** Average fluorescence intensity of JC-1 monomer in each group of cells. **E** Schematic diagram of DNA damage experiment. **F** 4T1 cells were incubated with DOX, ICG and DIMP, and then DNA damage was observed with or without laser treatment. γ-H2AX (green) and DAPI (blue) were used for staining and marking (Scale bar: 20 μm). **G** The average fluorescence intensity of γ-H2AX in each group. **H** Signal intensity distribution of DOX, DIMP, ICG + L, DIMP + L channels

As a cellular energy factory, mitochondria played vital roles in cell stability. The co-localization of lysosome and mitochondria was observed by CLSM. As shown in Fig. 3A, the Mander coefficient was 0.56 for DOX and 0.74 for ICG at 2 h. At 6 h, the Mander coefficient was significantly increased, which was 0.77 for DOX and 0.91 for ICG. The cellular fluorescence intensity was quantified (Fig. 3B) and the fluorescence intensity of DOX and ICG had a significant difference over time.

### Cell damage test

The cell damage was tested with JC-1 stained experiment for determining the change of mitochondrial transmembrane potential induced by DMIP. When the membrane potential of cells was normal, JC-1 entered into the mitochondrial matrix through the mitochondrial membrane and formed a polymer emitting red fluorescence due to the increased concentration. However, for the apoptotic cells, the membrane potential was low. JC-1 was released from the mitochondria, which could not be gathered in the mitochondrial matrix to form a monomer emitting green fluorescence. The results could be qualitative and quantitative by detecting green and red fluorescence. As shown in Fig. 3C, comparing with the control group, the green fluorescence of DIMP + L group was the strongest, and the red fluorescence was the weakest. Meanwhile, the green fluorescence of DIMP group was only weaker than that of DIMP + L group, and the red fluorescence was only stronger than that of DIMP + L Group. As shown in Fig. 3D, the average fluorescence of DIMP + L group was the strongest. These results indicated that our DIMP could decline mitochondrial membrane potential and promote apoptosis.

Previous experiments have proved that DIMP was well located in lysosomes. Due to the decomposition behavior of DIMP triggered in the acidic environment of lysosomes, acridine orange (AO) staining was performed to determine the damage of DIMP to lysosomes. As shown in Additional file 1: Fig. S5, bright red fluorescence was observed in intact lysosomes in untreated control cells, resulted from the accumulation of AO in acidic environment of lysosomes. After the treatment with DIMP + L, the red fluorescence disappeared and the cells shrank, indicating the destroyed lysosomes and apoptotic cancer cells.

The cellular DNA damage of drugs was studied with a step diagram (Fig. 3E). Though the CLSM image (Fig. 3F), it could be clearly seen that the green fluorescence intensity was stronger in the DIMP group and the DIMP + L group, indicating more accumulation of γ-H2AX. The fluorescence intensity was obtained quantitatively in image (Fig. 3G). It confirmed that the fluorescence intensity was strongest in DIMP + L group, with significant difference from other groups. A cell was randomly selected and quantified to obtain the fluorescence curve (Fig. 3H), and it further illustrated that the fluorescence intensity of the DIMP + L group was significantly higher than that of the other groups.

### Immunologic cell death effect by DIMP

**Fig. 4:**
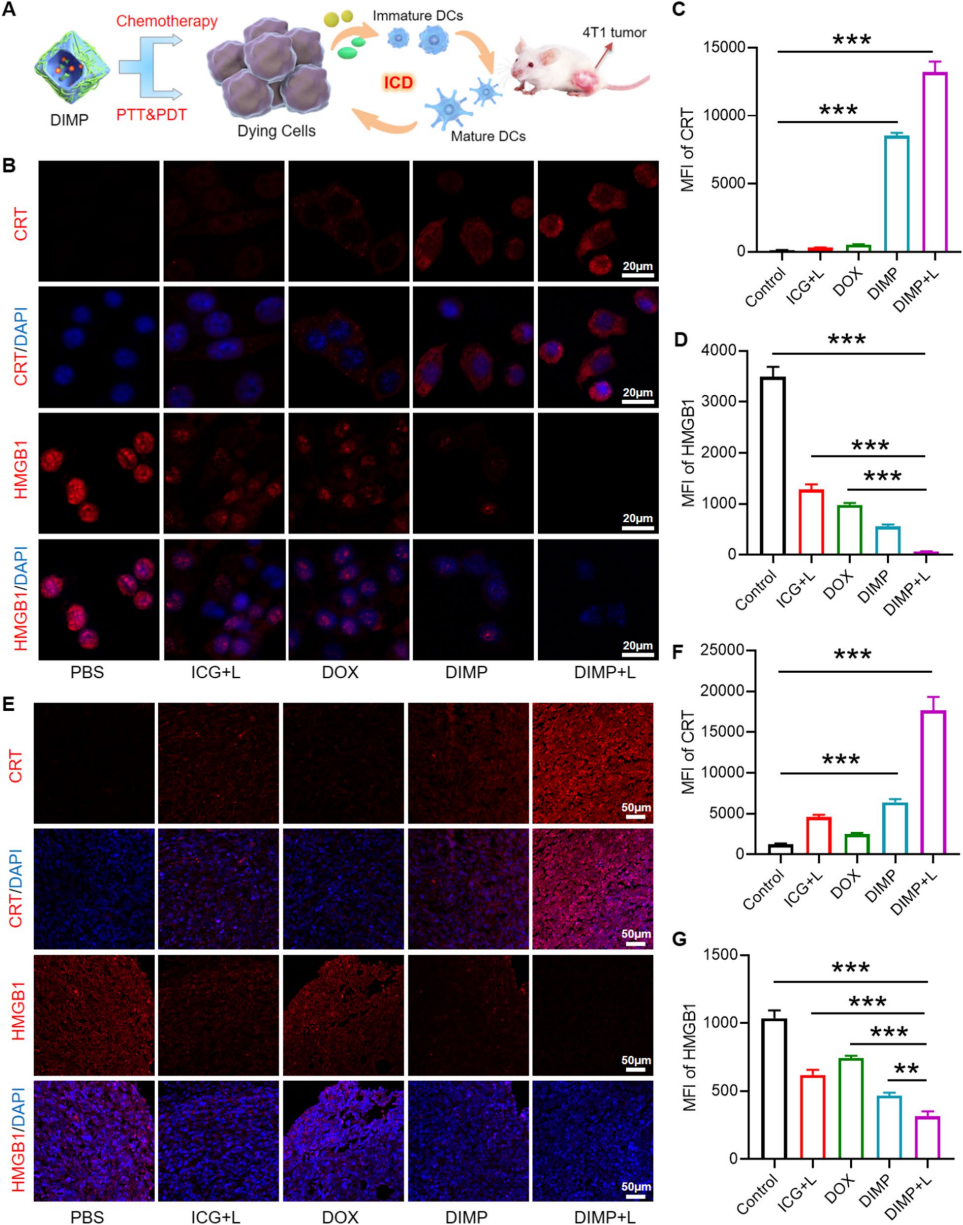
The Immunogenic cell death process induced by DIMP in vitro and in vivo (**A**). Representative confocal images and corresponding quantifications analysis of CRT and HMGB1 after treatment with PBS, ICG + L, DOX, DIMP and DIMP + L for 24 h in 4T1 cells (**B**–**D**) and in 4T1 tumor sections (**E**–**G**)

Conventional tumor therapies such as chemotherapy and phototherapy have been considered as effective tool for boosting immunogenic cell death (ICD) against a broad spectrum of solid tumors. Considering the powerful ability of combination therapy of DIMP, we further gained the results of biochemical correlates including calreticulin (CRT) and high-mobility group box 1 (HMGB1) in the extracellular milieu and in-vivo tumor sections (Fig. 4A). As showed in Fig. 4B–D, we could clearly witness the overexpression of CRT, which released a “eat me” signal and provided basics for the phagocytosis of DC cells. Compared with PBS group, all drug groups showed an obvious CRT expression, and the DIMP + L group exhibited greater effect than that of DIMP group, which was owing to the combined treatment of chemotherapy and PTT/PDT for the former. Meanwhile, the HMGB1 protein could be obviously spread to the extracellular matrix from cell nucleus, which could promote the maturation process of dendritic cells (DCs). Further, the ICD process was also carried out in tumor tissue (Fig. 4E–G), and the similar results to cell level were obtained. After irradiation with laser (808 nm), the DIMP + L group generated forceful ICD effect. All these excellent results collectively validated that DIMP could be served as outstanding ICD-inducer for rousing immune response.

### Tumor penetration

**Fig. 5:**
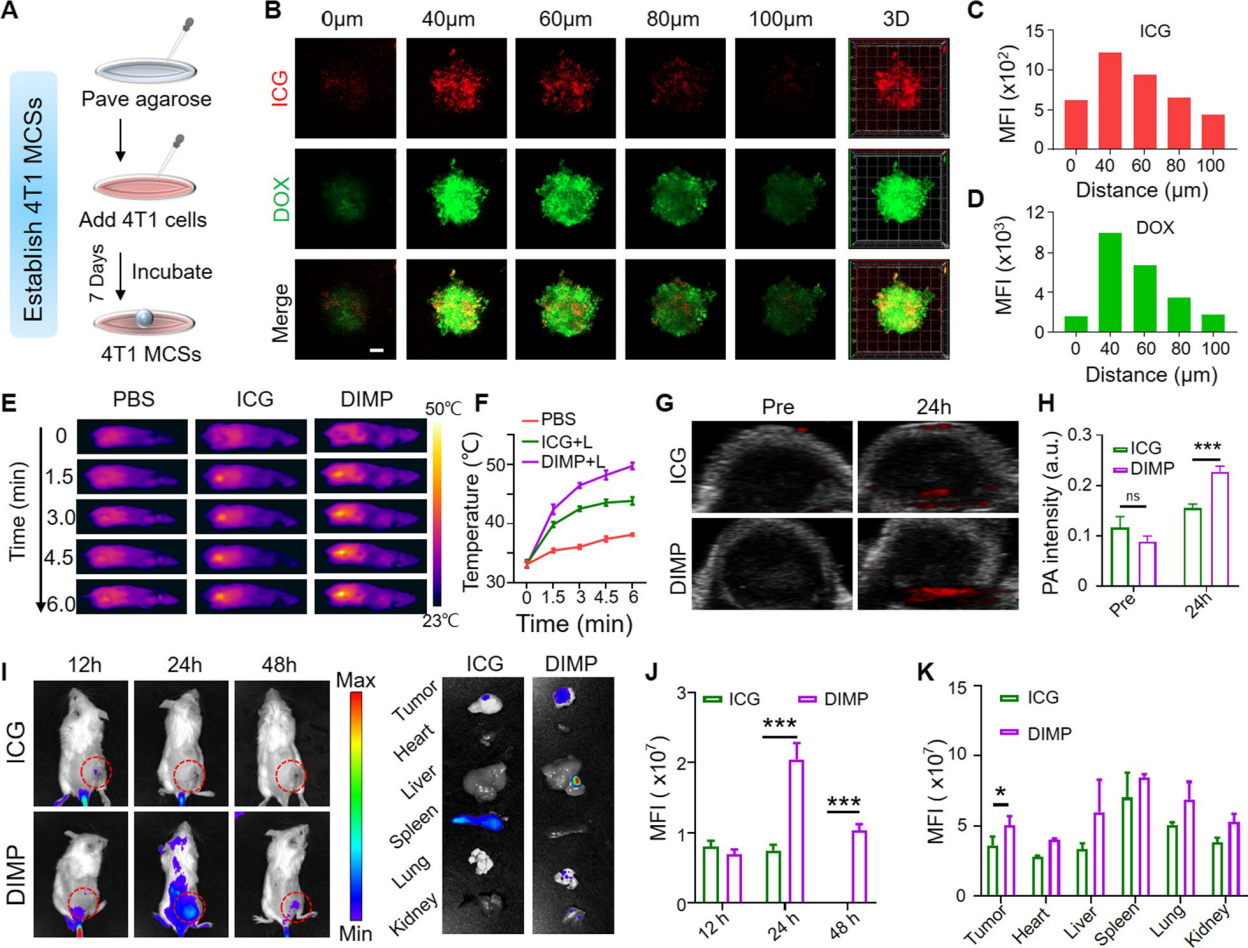
**A** Schematic diagram of tumor penetration with 4T1 MCSs. **B** 4T1 MCSs were incubated with DIMP at 37 °C for 6 h and observed with laser confocal microscopy. **C**, **D** The average fluorescence intensity of ICG and DOX at different depths (Scale bar: 20 μm). **E** IR thermal imaging of mice treated with ICG and DIMP at different time points the corresponding temperature change **F**. **G** In vivo PA imaging of tumor in tumor-bearing mice 24 h after intravenous injection of DIMP. **H** Changes of PA signal intensity of DOX and ICG in tumor area. **I** Fluorescence images of 4T1 tumor-bearing mice injected with ICG and DIMP at different time points (tumors were highlighted by red circles), in vivo fluorescence images of tumors and organs (heart, liver, kidney, spleen and lung). **J** Quantification of the fluorescence intensity of the tumor at different time points. **K** Quantification of the fluorescence intensity of tumors and organs (heart, liver, kidney, spleen and lung)

The size and shape of nano-carriers made important effects on the tissue infiltration of anti-tumor drug [41, 42]. In order to simulate the tumor penetration of DIMP, MCSs of 4T1 cells were used as an in vitro model. The MCSs was established (Fig. 5A) and incubated with DIMP (containing 20 μg/mL DOX) for 8 h (Fig. 5B). The DOX with green fluorescence and ICG with red fluorescence were observed and quantified on MCSs (Fig. 5C, D). It could be found that the fluorescence intensity of DOX and ICG was the strongest when the scanning depth reached 40 μm, respectively. And the fluorescence signal intensity distribution of the MCS was analyzed in Additional file 1: Fig. S6. It proved that DIMP could be enriched in tumor site with good permeability.

### In vivo bio-distribution analysis

As a clinical NIR dye, ICG can efficiently absorb NIR light and convert it into heat. Under the irradiation of 808 nm laser, the temperature change of the whole body of the mouse was recorded by a thermal imager. As shown in the Fig. 5E, with the increase of laser irradiation time, the temperature of the PBS group did not change significantly, while the temperature of the ICG group was gradually increased. It was obvious that the temperature of the tumor site in the DIMP group was increased significantly. The thermal imager recorded the temperature increase over time. The temperature of tumor was recorded (Fig. 5F), which could be as high as 50 °C within 6 min of irradiation. It proved that ICG could be enriched in the tumor thus making excellent photothermal effects.

As a new type of biomedical imaging method, PA imaging could obtain high-resolution and high-contrast tissue fluorescence signal images. As an effective contrast agent for PA, ICG was conductive to analyze the fluorescent signal distribution of DIMP in tumor models. A mouse tumor model was used to verify the performance of DIMP in vivo. The fluorescence imaging results suggested that the drug was enriched in the tumor site within 24 h after drug injection (Fig. 5G). The DIMP group had obvious PA signal, which was stronger than that of ICG group. Above conclusion was verified with quantitative analysis (Fig. 5H). In general, DIMP could be accumulated in tumor tissues and applied for imaging, which could provide accurate information on tumor micro-structure.

Based on the conclusions of above in vivo experiments, 24 h after drug injection was regarded as the best time point for laser irradiation. The fluorescence distribution at 12 h, 24 h, and 48 h after the injection of the drug (Fig. 5I). For the ICG group, the drug could reach the tumor site at 12 h, and then the fluorescence was decreased at 24 h, and almost no fluorescence could be observed at 48 h. For the DIMP group, the drug began to gather at the tumor site at 12 h, and fluorescence was observed throughout the body at 24 h, mainly at the tumor site. At 48 h, the drug only gathered at the tumor site. At 48 h, the main organs of the mice were dissociated for fluorescence scanning. The fluorescence was almost invisible in ICG group, which may be resulted from the metabolism of drugs. In the DIMP group, a part of the fluorescence could be still observed in the tumor site, and little fluorescence was found in other organs. This may be because that the drug was cleared through the blood circulation. The fluorescence quantification results of both tumors and major organs by IVIS imaging software also verified above conclusions (Fig. 5J, K). CLSM of the dissociated tumor tissue slices could also show that the DIMP group had higher DOX and ICG fluorescence signals (Additional file 1: Fig. S7). It suggested that DIMP had excellent permeability and enhanced permeability and retention (EPR) effects, which could be efficiently gathered at the tumor site, and be not easily cleared by the blood circulation. The above results brought confidence for the further in vivo experiments.

### In vivo anti-cancer activity

**Fig. 6:**
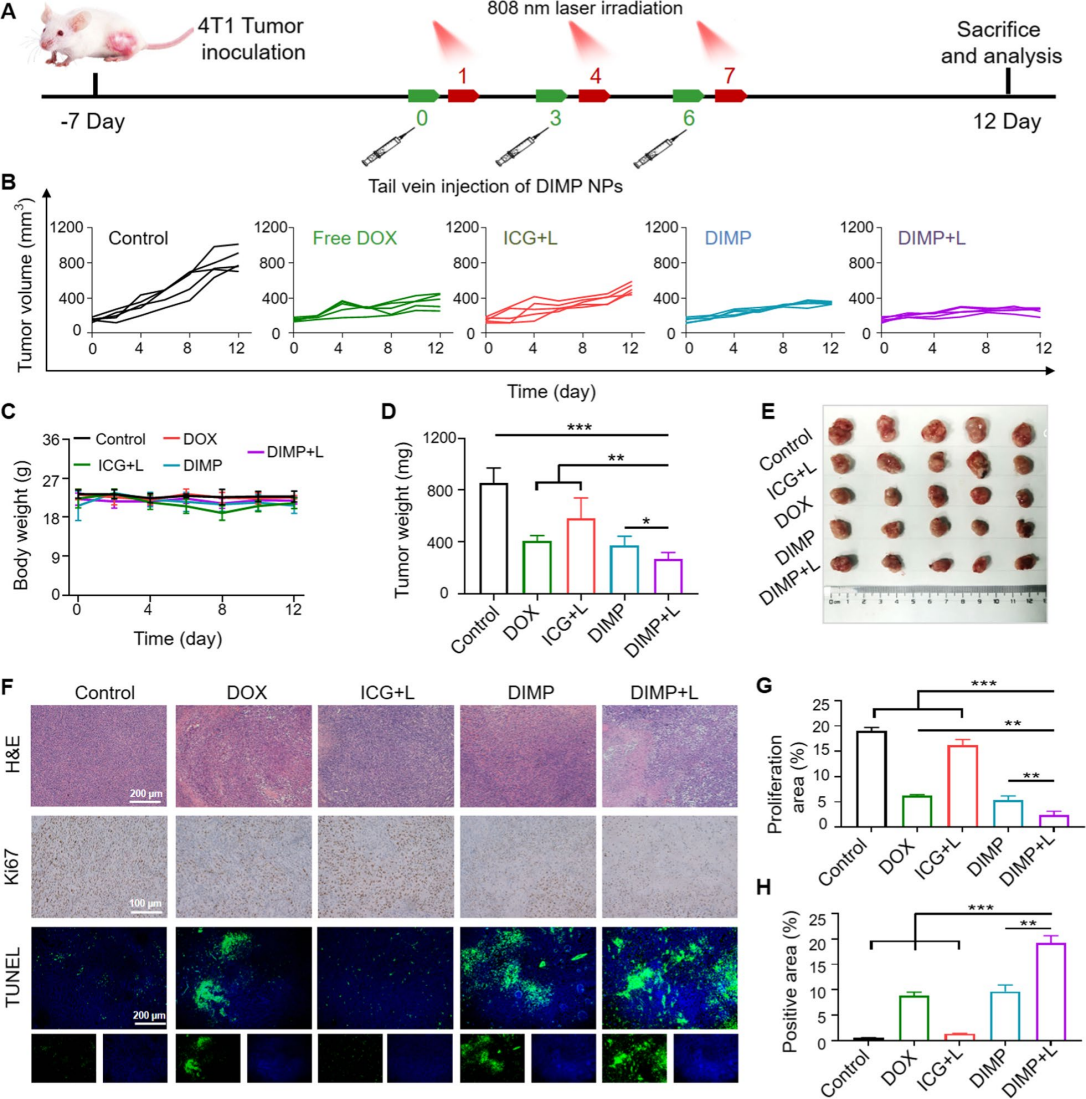
**A** Schematic illustration of the treatment process of DIMP. **B** Tumor growth curves of mice under different treatment conditions (n = 5). **C** During the treatment, the body weight change curve of each group. **D** Tumor weight and **E** photos of mice in each group after treatment. **F** H&E, Ki67 and TUNEL stained sections of tumors in different groups. Quantitative analysis of **G** Ki67 and **H** TUNEL in sections of tumor tissue

Encouraged by above results, the 12-day in vivo treatment experiment was quickly launched (Fig. 6A). Three times of tail vein injection were required, and light was given 24 h after each injection. The DIMP group performed well in tumor treatment (Fig. 6B), and the tumor size in the DIMP + L group showed a steady trend of decline. It showed the excellent anti-tumor effects of DIMP.

During the treatment, the body weight of the mice was recorded daily (Fig. 6C), and the body weight of the mice in each group maintained stable without significant changes. The dissociated mice tumors were weighed (Fig. 6D) and photographed (Fig. 6E). The weight of the tumor in the DIMP + L group was the smallest with a significant difference from other groups, which further illustrated the excellent anti-tumor effects in DIMP + L group.

The main organs were sliced and stained with H&E, Ki67 and TUNEL. The H&E results (Fig. 6F and Additional file 1: Fig. S9) showed no obvious lesion. As shown in Fig. 6G, after calculation, the number of proliferation factors in the DIMP + L group was significantly different from that of other groups. In Fig. 6H, TUNEL results were quantified using image J software, indicating more apoptotic area in DIMP + L group. The tumor tissues of the treatment group were sliced to observe the drug fluorescence, and the DIMP + L group also showed stronger drug fluorescence than free drugs. It showed that DIMP had excellent anti-tumor effects in vivo.

### Biosafety analysis

**Fig. 7:**
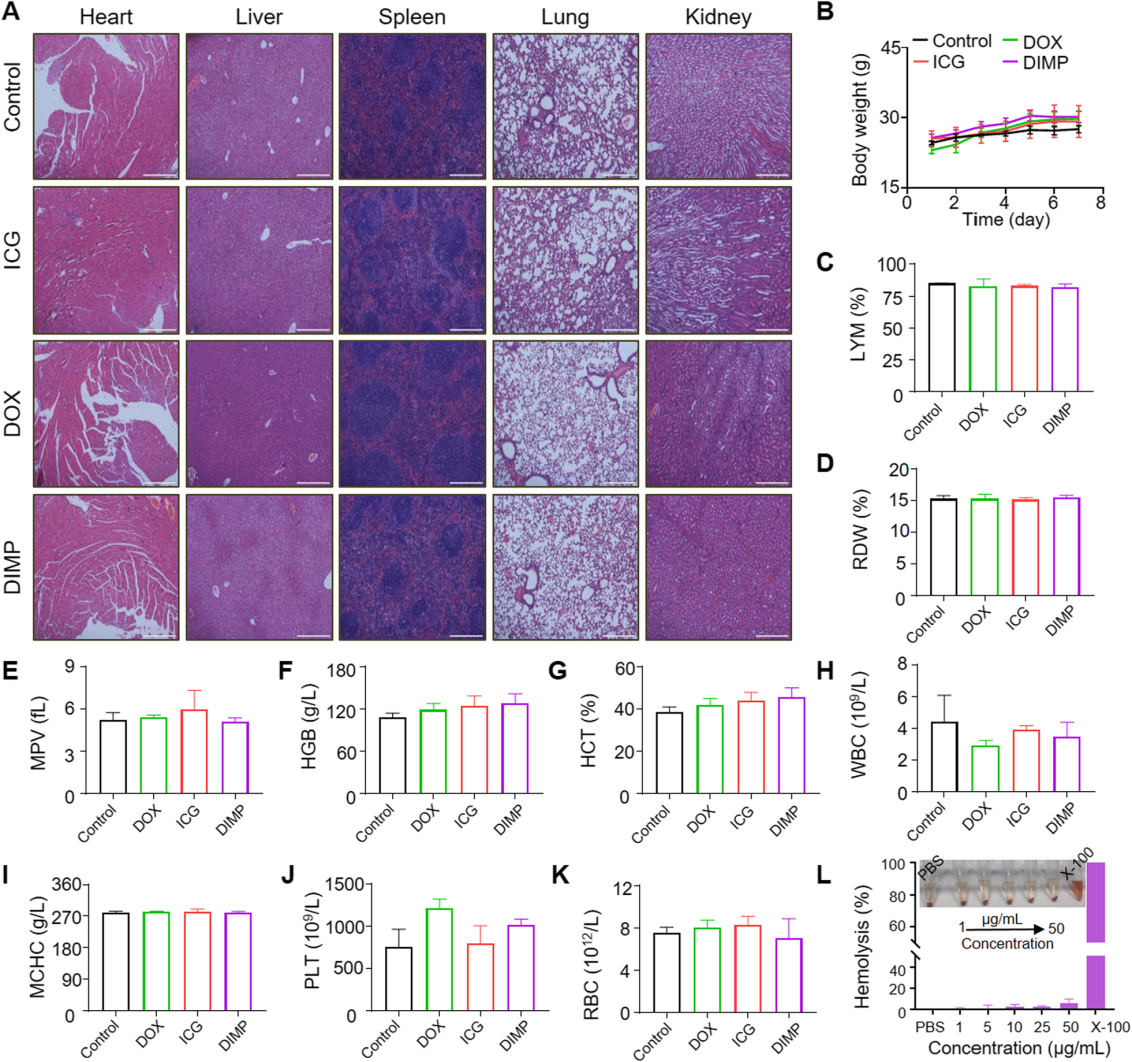
**A** H&E staining of main organs of mice after different treatments (Scale bars: 200 μm). **B** Body weight changes of Balb/c mice within 7 days after injection of PBS, ICG, DOX and DIMP. **C**–**K** Blood parameters of Balb/c mice 7 days after injection of PBS, ICG, DOX and DIMP. Data were expressed as mean ± SD (n = 3). **L** Photos of hemolysis and hemolysis rate after treatment with different concentrations of DIMP

In order to evaluate the in vivo biosafety of DIMP, healthy Balb/c mice were injected with Control (PBS), DOX, ICG and DIMP via tail vein. After 7 days, the heart, liver, spleen, lung and kidney of mice were taken and sliced for H&E staining. As showed in Fig. 7A, the DOX group showed a cardiotoxicity [43] and the DIMP could effectively ameliorate this phenomenon. Furthermore, as shown in Fig. 7B, the weight of the mice did not change significantly within 7 days. Further, whole blood was collected for hematological analysis (Fig. 7C–K), involving several parameters including WBC, RBC, MCHC, RDW, LYM, HCT, HGB, MPV and PTL etc. Comparing with the control group, there was no significant difference in all these parameters. The DIMP NPs (at concentration of 1, 5, 10, 25, 50 μg/mL) were mixed with the blood and the leaked hemoglobin was detected at 570 nm by UV–Vis absorption spectra (Additional file 1: Fig. S10). Both the DIMP NPs and PBS control showed a leakage of less than 5% (Fig. 7I), suggesting a good biological safety.

In summary, we have successfully developed an intracellular acidity-responsive MOF nanoreactor of DIMP for cancer therapy. The well-designed DIMP presented high drug loading capacity, controlled drug release, low side effects and good tumor suppression effect. After laser irradiation, ICG in DIMP could not only produce ROS, but also elevated temperature at the tumor site, exhibiting improved efficacy than that of free ICG. Further, DIMP was armed with a multi-mode FL/IR/PA imaging and a combined PTT/PDT/chemotherapy for effectively inhibiting the growth of 4T1 tumors in vivo. These results proved the potential therapeutic value of DIMP in cancer theranostics, and may provide a new strategy for the establishment of an integrated nanoplatform for tumor diagnosis and treatment.

## Supplementary Information

**Additional file 1: Figure S1. **XRD results of ZIF-8 and DIMP. **Figure S2. **XPS pattern including full survey spectrum of the prepared DIMP. **Figure S3.** Under different time periods of 808 nm laser irradiation, the absorption spectra of DPBF changes in the presence of DIMP. **Figure S4. **The cells were incubated with DIMP for 2 h and 6 h to obtain CLSM images of lysosome colocalization. Scale bar: 50 μm. **Figure S5. **AO staining method showed that DIMP had acid-triggered decomposition properties and lysosomal destruction. Scale bar: 50 μm. **Figure S6.** Corresponding to the fluorescence signal intensity distribution of the MCSs. **Figure S7.** Tumor slice images after 48 h injection of ICG, DOX and DIMP. **Figure S8. **Tumor slice images after treatment with ICG, DOX, DIMP and DIMP + L, Scale bar = 200 µm. **Figure S9. **H&E stained sections of mice heart, liver, spleen, lung and kidney after 7 days’ injection of Control, DOX, ICG + L, DIMP and DIMP + L. **Figure S10. **The UV–Vis absorption spectra of red blood cells were treated with DIMP at different concentrations. (Triton X-100 group was the positive group and PBS group was the negative group).

## Data Availability

The data of this study is available from the corresponding authors on reasonable request.
