# Supplementary material for: Acidic microenvironment responsive polymeric MOF-based nanoparticles induce immunogenic cell death for combined cancer therapy
Source: J Nanobiotechnology. 2021 Dec 28;19:455. doi: 10.1186/s12951-021-01217-4 (PMC8715615; doi:10.1186/s12951-021-01217-4)
Supplement: Supplementary file 1 — Additional file 1: Figure S1. XRD results of ZIF-8 and DIMP. Figure S2. XPS pattern including full survey spectrum of the prepared DIMP. Figure S3. Under different time periods of 808 nm laser irradiation, the absorption spectra of DPBF changes in the presence of DIMP. Figure S4. The cells were incubated with DIMP for 2 h and 6 h to obtain CLSM images of lysosome colocalization. Scale bar: 50 μm. Figure S5. AO staining method showed that DIMP had acid-triggered decomposition properties and lysosomal destruction. Scale bar: 50 μm. Figure S6. Corresponding to the fluorescence signal intensity distribution of the MCSs. Figure S7. Tumor slice images after 48 h injection of ICG, DOX and DIMP. Figure S8. Tumor slice images after treatment with ICG, DOX, DIMP and DIMP + L, Scale bar = 200 µm. Figure S9. H&E stained sections of mice heart, liver, spleen, lung and kidney after 7 days’ injection of Control, DOX, ICG + L, DIMP and DIMP + L. Figure S10. The UV–Vis absorption spectra of red blood cells were treated with DIMP at different concentrations. (Triton X-100 group was the positive group and PBS group was the negative group). [file 12951_2021_1217_MOESM1_ESM.docx]

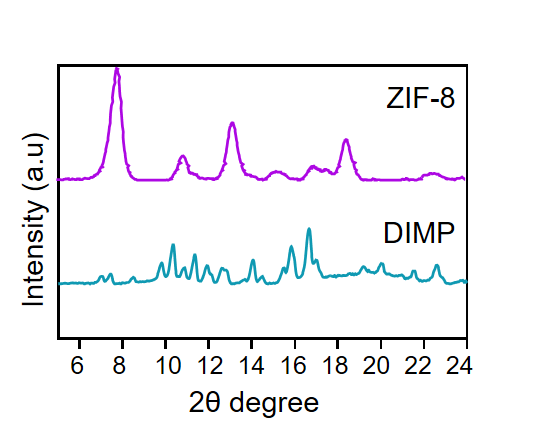


**Figure S1.** XRD results of ZIF-8 and DIMP.


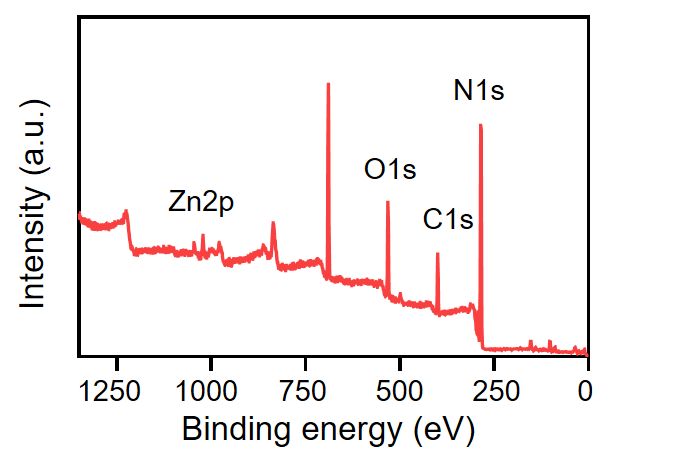


**Figure S2.** XPS pattern including full survey spectrum of the prepared DIMP.


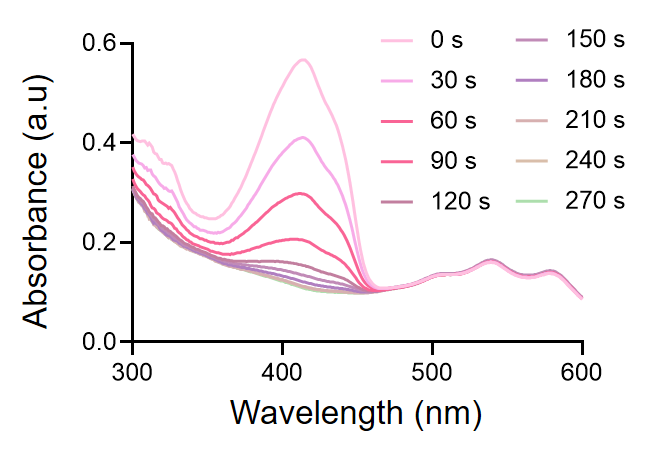


**Figure S3.** Under different time periods of 808 nm laser irradiation, the absorption spectra of DPBF changes in the presence of DIMP.


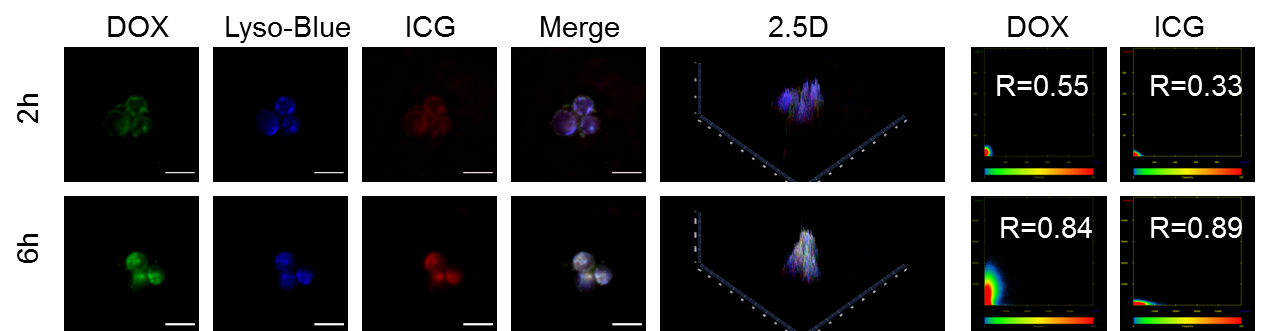


**Figure S4.** The cells were incubated with DIMP for 2 h and 6 h to obtain CLSM images of lysosome colocalization. Scale bar: 50 μm.


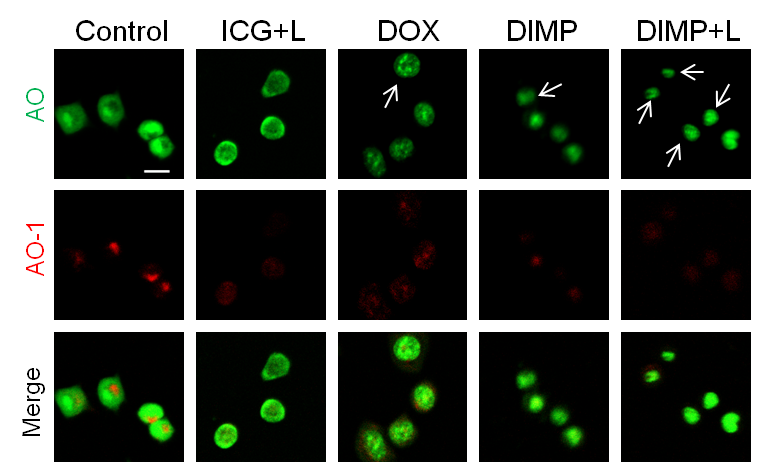


**Figure S5.** AO staining method showed that DIMP had acid-triggered decomposition properties and lysosomal destruction. Scale bar: 50 μm.


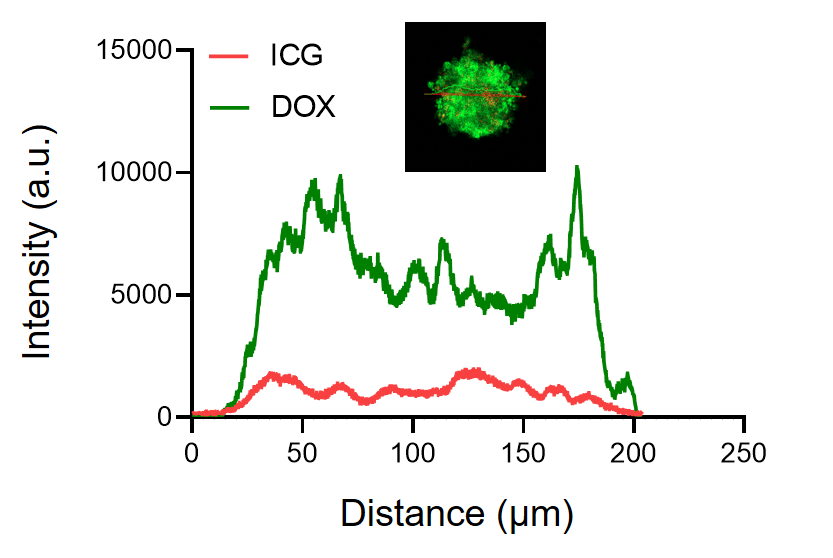


**Figure S6.** Corresponding to the fluorescence signal intensity distribution of the MCSs.


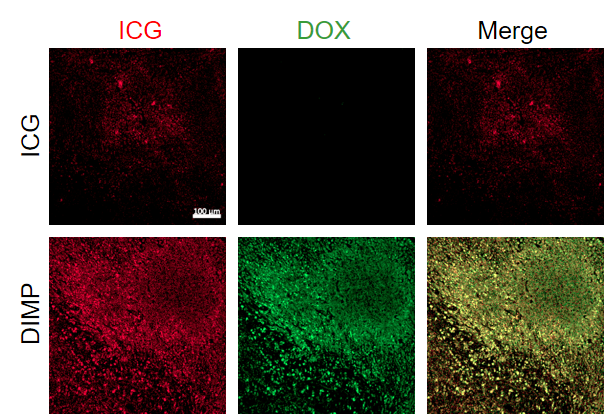


**Figure S7.** Tumor slice images after 48 h injection of ICG, DOX and DIMP.


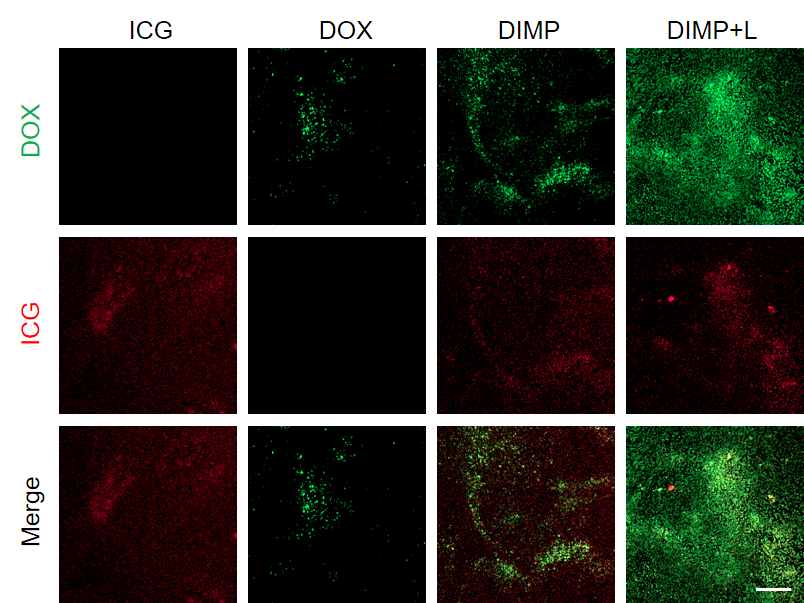


**Figure S8.** Tumor slice images after treatment with ICG, DOX, DIMP and DIMP+L, Scale bar = 200µm.


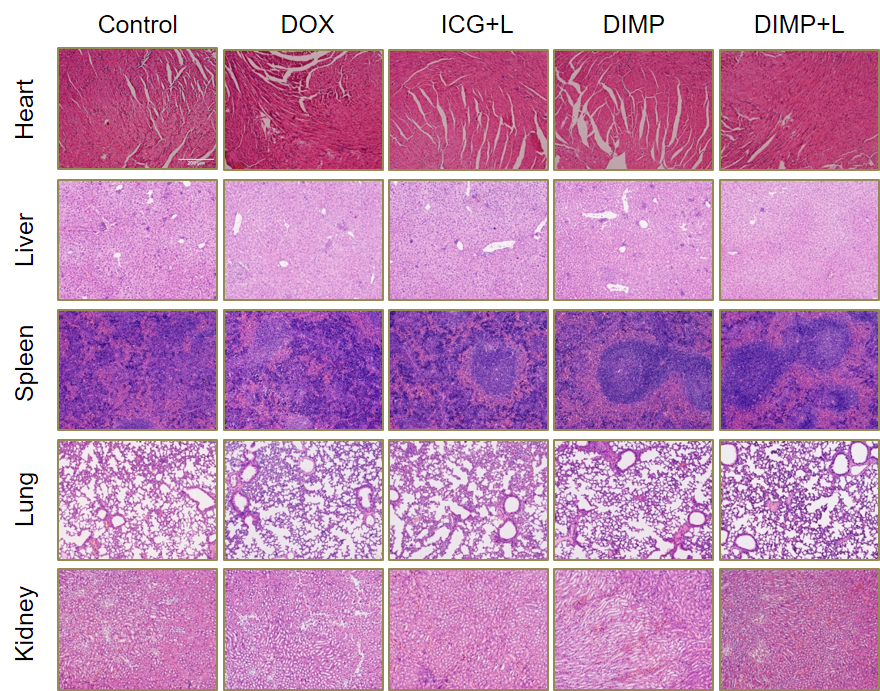


**Figure S9.** H&E stained sections of mice heart, liver, spleen, lung and kidney after 7 days’ injection of Control, DOX, ICG+L, DIMP and DIMP+L.


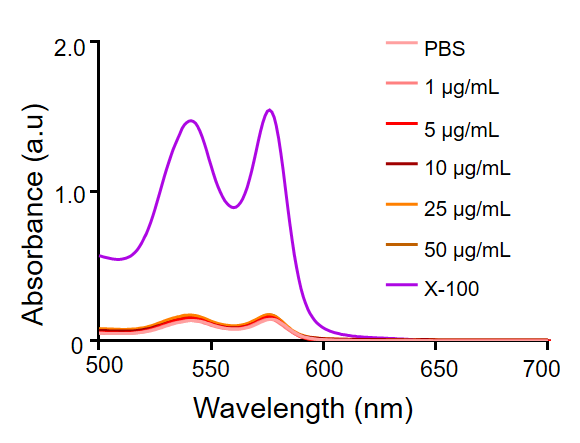


**Figure S10.** The UV-Vis absorption spectra of red blood cells were treated with DIMP at different concentrations. (Triton X-100 group was the positive group and PBS group was the negative group).
